# Seasonal variation in dietary diversity and food variety scores among an indigenous Karen population in western Thailand: a cross-sectional study

**DOI:** 10.1017/S1368980025101225

**Published:** 2025-09-25

**Authors:** Pattamaporn Joompa, Sueppong Gowachirapant, Sinee Chotiboriboon, Rungnapha Sarasak, Nattapach Thongkam, Prapa Kongpunya, Wantanee Kriengsinyos

**Affiliations:** 1 Institute of Nutrition, Mahidol University, Nakhon Pathom, Thailand; 2 Research Center for Nutrition Support, Faculty of Medicine Siriraj Hospital, Mahidol University, Bangkok, Thailand

**Keywords:** Dietary diversity, School-age children, Working-age people, Older people, Indigenous people

## Abstract

**Objective:**

This study compared dietary diversity and nutrient adequacy across age groups and seasons within an indigenous Karen community.

**Design:**

Cross-sectional survey.

**Setting:**

Dietary intake was assessed using a single-day 24-h dietary recall among Karen community members living in two villages of Laiwo subdistrict, Sangkhlaburi district, Kanchanaburi province, Thailand.

**Participants:**

In total, 312 Karen people participated during the rainy season and 344 during the dry season, including school-age children (6–12 years), working-age people (19–59 years) and older people (≥ 60 years).

**Results:**

Dietary diversity scores and food variety scores significantly differed across age groups for both seasons. However, seasonal dietary diversity score differences were not observed within any group, except for the food variety scores of school-age children. Over 70 % of participants in all age groups had inadequate intake of key micronutrients – Ca, Fe, vitamin A, vitamin C, Zn, vitamin B_6_ and vitamin B_12_ – as indicated by nutrient adequacy ratios < 0·75. Moderate to strong positive correlations between dietary diversity scores and nutrient adequacy ratios for energy, vitamin B_2_, vitamin C, niacin and mean adequacy ratio (*r* = 0·418–0·691, *P* < 0·001) were observed exclusively in the dry season and across all age groups.

**Conclusions:**

Among the Karen people, who are also facing triple burden malnutrition, dietary diversity is limited, micronutrient inadequacy is prevalent and overall dietary quality is insufficient despite frequent vegetable consumption. Findings highlight the need to address systemic challenges related to food variety and to promote education on appropriate food quantities, preparation methods and sustainable traditional food systems to improve nutrition.

Malnutrition remains a persistent global health and development concern and is a major barrier to achieving Sustainable Development Goals by 2030. It reflects critical shortcomings in both health and food security systems and is driven by a complex interplay of factors including inadequate food quantity, poor dietary quality and socio-economic inequality^(1)^. Ensuring nutritional adequacy is essential throughout the life course, as nutrient needs vary with age and physiological changes. A diet characterised by diversity and nutrient density is widely recommended to meet these dynamic needs^(2)^. However, imbalanced intake and impaired nutrient absorption can lead to persistent forms of malnutrition, most notably micronutrient deficiencies – particularly of Fe, riboflavin, folate and vitamin C – which remain prevalent globally and thus highlight the need to improve overall dietary quality^(3)^.

Currently, several upper-middle-income countries are facing a triple burden of malnutrition, marked by the simultaneous presence of overnutrition, undernutrition and micronutrient deficiencies^(4)^. Evidence from the South East Asian Nutrition Surveys II revealed that this nutritional complexity is increasingly evident across Thailand’s population. While stunting remains prevalent primarily among infants and toddlers, the prevalence of overweight and obesity has increased significantly with age. Concurrently, inadequate intake of essential micronutrients – including Ca, Fe, Zn and vitamins A, C and D – was found to be alarmingly high, affecting between 53·2 and 93·6 % of individuals assessed^(4)^. These findings highlight the need for age-sensitive and context-specific strategies to address both nutritional deficits and excesses.

A key driver of this evolving nutritional landscape is an ongoing nutrition transition, characterised by a shift from traditional diets, which primarily rely on homegrown or foraged foods, to market-based diets rich in energy-dense and processed foods^(5)^. This shift has contributed to dietary imbalances, with increased intake of calories but reduced intake of essential nutrients. The effects of this transition are particularly profound among indigenous ethnic populations, whose food systems are closely intertwined with cultural identity, environmental sustainability and local biodiversity. As these communities become increasingly integrated into a market economy, traditional food practices are disrupted and nutritional vulnerability increases, exacerbated by limited access to diverse, nutrient-rich foods^(6)^. Indigenous peoples also often face structural inequities, including limited access to basic healthcare and public services, which leads to their underrepresentation in national health statistics and research^(7)^. Beyond systemic marginalisation, seasonal fluctuations in food availability – driven by environmental cycles and climatic conditions – further compound the risks of food insecurity and malnutrition among these communities^(2,8)^.

In Thailand, the Karen people, the largest indigenous minority ethnic group, reside in rural and mountainous regions across the northern, central and western provinces^(9)^. Due to the geographical isolation of their settlements and the predominant reliance on rotational farming alongside other agricultural practices, the Karen people remain largely dependent on localised, community-based food systems^(10,11)^. Rooted in biodiversity, indigenous knowledge and traditional wisdom, these food systems support a wide variety of food sources and culinary practices^(11)^. However, socio-economic transformation, climate change, rural-to-urban migration and the growing influence of modern dietary patterns have increasingly disrupted these systems. Consequently, changes in diet have contributed to lower nutrient intake and a rise in non-communicable diseases^(11)^.

Although Thailand’s Department of Health has established the Health Center for Ethnic Groups, Marginalized People, and Migrant Workers strategy to address health disparities at the policy level, comprehensive and disaggregated data on indigenous populations remain limited. While some studies have examined the food consumption behaviours of groups such as the Karen and Akha^(12,13)^, there remains a lack of clear research that explores the unique dietary patterns shaped by regional, ethnic and cultural contexts – patterns that are crucial for understanding the nutritional challenges and needs of these communities^(14)^.

To assess nutritional adequacy in diverse populations, the dietary diversity score (DDS) has been widely employed as a practical tool for assessing dietary quality. It is recognised for its simplicity, cost-effectiveness and applicability across various populations and age groups^(15,16)^. DDS is calculated based on the number of food groups consumed over a specific reference period, typically the preceding 24 h, with higher DDS values generally indicating better dietary quality^(15,16)^. In addition, the food variety score (FVS), which measures the number of individual food items consumed, serves as an additional non-invasive indicator of nutritional adequacy^(17)^. Both DDS and FVS have shown significant associations with nutritional adequacy across different age groups^(18,19)^. These user-friendly and economical tools are extensively applied in nutritional assessments, particularly in resource-limited settings, and are especially relevant for evaluating vulnerable populations such as children, the elderly, pregnant women, indigenous groups and migrant communities^(20)^.

Accordingly, this study aimed to assess dietary diversity, food variety and nutrient adequacy among the Karen people across three age groups – school-age children, working-age people and older people – by comparing dietary intake between the rainy and dry seasons. This study also examined the correlation between the DDS and both the nutrient adequacy ratio (NAR) and the mean adequacy ratio (MAR). The findings are expected to inform the development of context-specific nutritional intervention strategies grounded in traditional food systems and local food security considerations.

## Methods

### Study design

This study is part of an international joint research project entitled Climate Change Resilience of Indigenous Socioecological Systems (RISE)^(21)^. A cross-sectional study was conducted in Laiwo subdistrict, Sangkhlaburi district, Kanchanaburi province, Thailand. This location is in Thungyai Naresuan National Wildlife Sanctuary in western Thailand, adjacent to the Myanmar border. Laiwo subdistrict consists of six Pwo Karen villages with similar lifestyles. Accessing these villages is quite difficult. During the dry season, they can be reached by a four-wheel drive vehicle, but during the rainy season, they can only be reached by motorcycle or on foot. Consequently, only two villages near the district centre were purposively selected for data collection.

### Participants

Participants were categorised into three age groups: school-age children (6–12 years), working-age people (19–59 years) and older people (≥ 60 years). Population data for the two villages were collected in 2021 by public health volunteers, community leaders and the local health-promoting hospital. The sample size for each village was calculated using the Taro Yamane formula^(22)^. After calculation and assuming a dropout rate of approximately 10 %, a total of 78 school-age children, 300 working-age people and 34 older people from the two villages were enrolled in this study.

Following the compilation of name lists, eligible participants were selected through simple random sampling for both villages. Inclusion and exclusion criteria were applied to ensure appropriate participant recruitment. Inclusion criteria were (1) school-age children, working-age people or older people who reside regularly in the two villages; (2) no history of mental illness as diagnosed by a doctor; and (3) neither serious medical conditions had been diagnosed by a doctor, nor a therapeutic diet was advised. Exclusion criteria were (1) participants with serious illnesses, such as an accident or symptoms arising from a chronic disease or other diseases during the period of data collection, and (2) participants who travelled outside the villages during the period of data collection.

### Anthropometric measurements

Body weight and height were measured for all participant age groups following a standardised anthropometric procedure^(23,24)^. Weight was measured using a bioelectrical impedance analysis (Tanita model BC-582), while height was measured using a portable stadiometer (Seca model 217). For school-age children (6–12 years), anthropometric status was determined according to the WHO growth reference for ages 5–19 years^(25)^. Z-scores for BMI-for-age, height-for-age and weight-for-age were calculated using the WHO AnthroPlus version 1.0.4 software^(26)^. Underweight (5–10 years), stunting (≥ 5 years) and wasting (≥ 5 years) were defined as weight-for-age < –2 sd, height-for-age < –2 sd and BMI-for-age < –2 sd, respectively. Overweight and obesity of children aged 5 years and above were defined as BMI-for-age > 1 sd to ≤ 2 sd and BMI-for-age > 2 sd, respectively. For working-age people (19–59 years) and older people (≥ 60 years), BMI was calculated and categorised according to WHO classifications: underweight (< 18·5 kg/m^2^), normal weight (18·5–24·9 kg/m^2^), overweight (25–29·9 kg/m^2^) and obese (≥ 30 kg/m^2^)^(27)^.

### 24-h dietary recall

Dietary intake was assessed using a single-day 24-h dietary recall^(28)^. A pilot test was conducted with non-participating community members from similar food environments to ensure the tool’s contextual relevance. Data were collected in two seasons: the rainy season (August 2022) and the dry season (February 2023). Trained researchers from the Institute of Nutrition, Mahidol University, conducted face-to-face interviews, during which participants were asked to recall all foods and non-alcoholic beverages consumed in the preceding 24 h. Information collected included food types, ingredients and estimated portion sizes. Local research assistants assisted with translation between the Thai and Karen languages to ensure clear communication. Guardians provided dietary information for school-age children when needed, including details on food preparation. School lunch menus – typically consisting of a main dish, fruit and milk – were reviewed to verify food items that may have been omitted by child participants. To support recall accuracy, a food photo book was utilised, containing 134 commonly consumed traditional foods, labelled in both the Thai and Karen languages. Nutritional information for food products purchased from local grocery stores was obtained from packaging labels.

### Dietary diversity and food groups

The DDS used in this study was adapted from the DDS endorsed by the FAO^(16)^ and the fifth Thai National Health Examination Survey to fit with the context of indigenous communities^(29)^. Dietary diversity was categorised into nine food groups based on an modified version of the DDS: (1) all starchy staples (cereals and tubers); (2) legumes, nuts and seeds; (3) vegetables; (4) fruits; (5) flesh foods; (6) fish and seafood; (7) dairy and dairy products; (8) fat and oil; and (9) non-alcoholic beverages (e.g. fruit juices, soft drinks, energy drinks, soy milk and other sweetened beverages). Consumption of ≥ 15 g/d from any food group was scored as 1 point; intake of < 15 g/d was scored as 0^(30)^. The total DDS ranged from 0 to 9, with higher scores indicating greater dietary diversity, as more food groups were consumed in adequate quantities (≥ 15 g/d per group). Additionally, an FVS was calculated as a simple count of individual food items consumed across the nine food groups.

### Nutrient intake and nutrient adequacy analysis

Daily nutrient intakes – including energy, protein, carbohydrates, fat and ten selected micronutrients (Ca, Fe, vitamin A, vitamin B_1_, vitamin B_2_, vitamin C, niacin, Zn, vitamin B_6_ and vitamin B_12_) – were estimated using INMUCAL-Nutrients V.4.0 software^(31)^. Nutrient adequacy was assessed as NAR, which reflect the sufficiency of an individual’s intake for specific nutrients. NAR were calculated for twelve nutrients: energy, protein, Ca, Fe, vitamin A, vitamin B_1_, vitamin B_2_, vitamin B_6_, vitamin B_12_, vitamin C, niacin and Zn. Each NAR was derived by dividing a participant’s daily intake of a given nutrient by its age- and sex-specific recommended nutrient intake^(32)^, based on the Thai Dietary Reference Intakes 2020^(33)^. The MAR was computed by averaging all NAR values^(32)^. NAR or MAR values below 0·75 were considered indicative of inadequate nutrient intake^(34–36)^. To assess daily energy intake, the percentage of energy contribution from carbohydrates, protein and fat was calculated and compared with the Acceptable Macronutrient Distribution Ranges: 45–65 % for carbohydrates, 20–35 % for fat and 10–35 % for protein^(37)^.

### Statistical analysis

Data analysis was performed using the SPSS version 21 statistical package (SPSS Corp.). Data normality was assessed using the Kolmogorov–Smirnov test. The continuous variables were presented as frequency, percentage or means with sd. The mean differences between groups were tested with one-way ANOVA or Student’s *t* test as required. The *χ* ^2^ test was used to compare NAR and MAR between seasons. The Spearman correlation coefficient (*r*) was used to measure the correlation between DDS and MAR. Statistical significance was considered at a two-tailed *P* < 0·05. Correlation strength was classified as strong (*r* ≥ 0·60), moderate (*r* = 0·40–0·59) and weak (*r* < 0·40)^(38,39)^.

## Results

### Anthropometric characteristics

A total of 59 school-age children, 185 working-age people and 40 older people from the two villages were assessed for body weight and height. The gender proportions between males and females were 26:33, 52:133 and 15:25 in school-age children, working-age people and older people, respectively. The mean ages of school-age children, working-age people and older people were 9·5 (sd 1·9), 40·5 (sd 11·2) and 67·5 (sd 8·0) years old, respectively. The mean weights and heights of school-age children, working-age people and older people were 33·1 (sd 14·5) kg, 134·3 (sd 13·5) cm; 57·5 (sd 12·8) kg, 154·3 (sd 6·9) cm; and 49·4 (sd 11·1) kg, 152·4 (sd 7·7) cm, respectively. Most school-age children had a normal nutritional status. However, 5·1 % were identified as stunted and thin, 8·5 % were overweight and 13·6 % were obese. Likewise, only 8·1 % of the working-age people were underweight. However, almost one-fourth of the working-age people were overweight (22·7 %), and more than one-tenth were obese (11·4 %). In the group of older people, approximately one-third were underweight (35·0 %), while more than one-tenth were overweight (12·5 %). Only 5 % were obese (Table 1).

**Table 1.** Nutritional status of participants (*n* 284)

	School-age children (*n* 59)		Working-age people (*n* 185)		Older people (*n* 40)	
	Mean	sd	Mean	sd	Mean	sd
Age, years	9·5	1·9	40·5	11·2	67·5	8·0
Gender						
Male:female, *n*:*n*	26:33		52:133		15:25	
Height, cm	134·3	13·5	154·3	6·9	152·4	7·7
Weight, kg	33·1	14·5	57·5	12·8	49·4	11·1
BMI, kg/m^2^	–		24·1	5·0	20·9	4·0
BAZ	0·1	1·4	–		–	
HAZ	−0·2	1·1	–		–	
WAZ (*n* 39, > 10 Y)	−0·1	1·4	–		–	
	*n*, %		*n*, %		*n*, %	
Stunting (*n*, %)	3, 5·1		–		–	
Wasting (*n*, %)	3, 5·1		–		–	
Underweight (*n*, %)	–		15, 8·1		14, 35·0	
Overweight (*n*, %)	5, 8·5		42, 22·7		5, 12·5	
Obesity (*n*, %)	8, 13·6		21, 11·4		2, 5·0	

Values are mean (sd).

BAZ, BMI-for-age.

WAZ, weight-for-age Z score.

HAZ, height-for-age Z score.

### Seasonal variation in dietary diversity score and food variety score

**Fig. 1:**
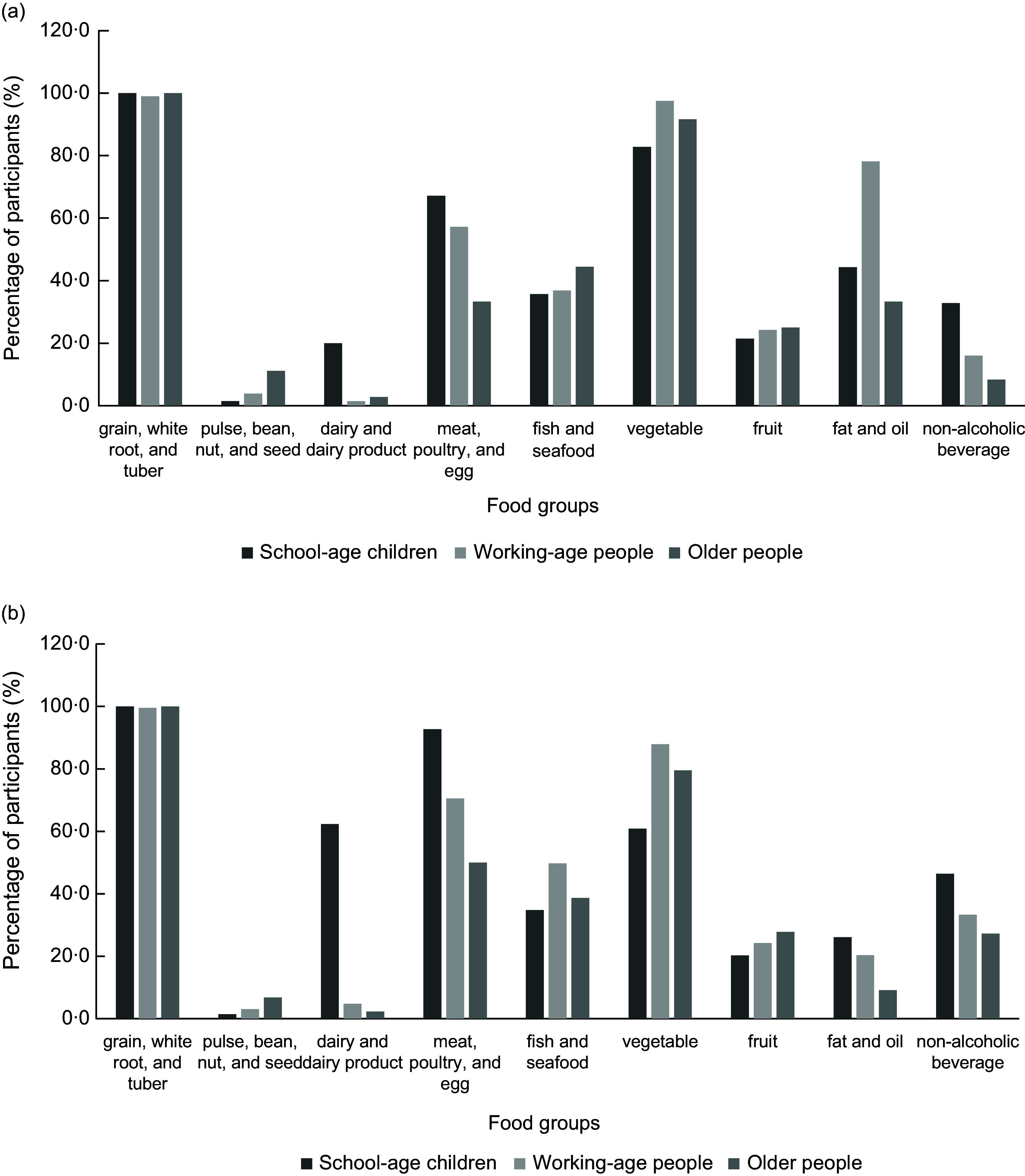
Percentage of participants in each age group consuming each food group during the rainy season (a) and the dry season (b).

The DDS among the three participant groups ranged from 3·39 to 4·09 (*P* < 0·05) during the rainy season and from 3·41 to 4·43 (*P* < 0·001) during the dry season. Similarly, the FVS ranged from 5·67 to 7·09 (*P* < 0·05) and from 5·41 to 8·17 (*P* < 0·001) in the rainy and dry seasons, respectively. Among all age groups, older people consistently exhibited the lowest DDS and FVS in both seasons (Table 2). Between the two seasons and within each participant group, only the FVS of school-age children showed a statistically significant difference (*P* < 0·05) (Table 3). During the rainy season, the five most commonly consumed food groups across all participant groups included (1) mainly rice (school-age children, 100 %; working-age people, 99·0 %; and older people, 100 %); (2) vegetables (school-age children, 82·9 %; working-age people, 97·6 %; and older people, 91·7 %); (3) meat, poultry and eggs (school-age children, 67·1 %; working-age people, 57·3 %; and older people, 33·3 %); (4) fat and oil (school-age children, 44·3 %; working-age people, 78·2 %; and older people, 33·3 %); and (5) fish and seafood (school-age children, 35·7 %; working-age people, 36·9 %; and older people, 44·4 %). However, the commonly consumed food groups during the dry season differed among the groups. Working-age people and older people mainly consumed (1) rice (working-age people, 99·6 %; older people, 100 %); (2) vegetables (working-age people, 87·9 %; older people, 79·5 %); (3) meat, poultry and eggs (working-age people, 70·6 %; older people, 50·0 %); and (4) fish and seafood (working-age people, 49·8 %; older people, 38·6 %). For the fifth most commonly consumed food group, working-age people reported a higher intake of non-alcoholic beverages (33·3 %), whereas older people consumed more fruits (27·8 %). In contrast, school-age children commonly consumed (1) rice (100 %); (2) meat, poultry and eggs (92·8 %); (3) dairy and dairy products (62·3 %), (4) vegetables (60·9 %); and (5) non-alcoholic beverages (46·4 %) (Fig. 1).

**Table 2.** Dietary diversity scores (DDS) and food variety scores (FVS) by participant group and season

	*n*	DDS		*P*	FVS		*P*
		Mean	sd		Mean	sd	
Rainy season							
School-age children	70	4·09	1·29	0·021^*^	6·90	3·08	0·042^*^
Working-age people	206	3·91	1·21		7·09	3·16	
Older people	36	3·39	1·20		5·67	2·83	
Dry season							
School-age children	69	4·43	1·14	< 0·001^*^	8·17	2·88	< 0·001^*^
Working-age people	231	3·93	1·17		6·93	3·26	
Older people	44	3·41	1·30		5·41	2·60	

Values are mean (sd).

*P* values were calculated using one-way ANOVA.

* *P* < 0·05.

**Table 3.** Comparison of dietary diversity scores (DDS) and food variety scores (FVS) between the rainy and dry seasons for each participant group

	DDS					FVS				
	Rainy		Dry		*P*	Rainy		Dry		*P*
	Mean	sd	Mean	sd		Mean	sd	Mean	sd	
School-age children	4·09	1·29	4·43	1·14	0·094	6·90	3·08	8·17	2·88	0·013^*^
Working-age people	3·91	1·21	3·93	1·17	0·840	7·09	3·16	6·93	3·26	0·601
Older people	3·39	1·20	3·41	1·30	0·943	5·67	2·83	5·41	2·60	0·673

*P* values were calculated using independent *t* test.

* *P* < 0·05.

### Nutrient adequacy assessment

NAR for all selected nutrients and MAR were calculated, with inadequacies identified using a cutoff of NAR and MAR < 0·75 (Table 4). Across both seasons, over two-thirds of the participants in all age groups had insufficient intakes of Ca, Fe, vitamin A, vitamin C, Zn, vitamin B_6_ and vitamin B_12_. Older people exhibited a broader range of nutrient inadequacies during both seasons compared with school-age children and working-age people. Among school-age children, significant seasonal differences were observed in the inadequacy of protein, vitamin B_2_, niacin and MAR. Among working-age people, significant differences between the rainy and dry seasons were found in the inadequate intake of energy, niacin, Zn, vitamin B_6_ and MAR. For older people, the only significant seasonal difference was Zn inadequacy. Energy intake and macronutrient distribution were also assessed (see online supplementary material, Supplemental Tables 2 and 3). The median energy intake of school-age children was 1194·2 kcal/d in the rainy season (% energy distribution of carbohydrate:protein:fat = 63·1:10·6:24·9) and 1240·5 kcal/d in the dry season (% of carbohydrate:protein:fat = 55·3:14·2:30·9). For working-age people, median energy intake was 1415·4 kcal/d during the rainy season (% of carbohydrate:protein:fat = 72·7:10·7:15·6) and 1094·3 kcal/d during the dry season (% of carbohydrate:protein:fat = 63·5:12·7:23·0). Among older people, energy intake was consistently low, with medians of 841·6 kcal/d in the rainy season (% of carbohydrate:protein:fat = 76·1:9·9:9·3) and 781·8 kcal/d in the dry season (% of carbohydrate:protein:fat = 68·5:10·5:17·2).

**Table 4.** Percentage of participants with inadequate nutrient intake (nutrient adequacy ratio and mean adequacy ratio (MAR) < 0·75)

Nutrient	School-age children					Working-age people					Older people				
	Rainy (*n* 70)		Dry (*n* 69)		*P*	Rainy (*n* 206)		Dry (*n* 231)		*P*	Rainy (*n* 36)		Dry (*n* 44)		*P*
	*n*	%	*n*	%		*n*	%	*n*	%		*n*	%	*n*	%	
Energy	38	54·3	34	49·3	0·674	111	53·9	156	67·5	0·003^*^	28	77·8	34	77·3	0·957
Protein	24	34·3	6	8·7	< 0·001^**^	110	87·4	140	60·6	0·128	27	75·0	37	84·1	0·312
Ca	69	98·6	67	97·1	0·551	180	80·1	201	87·0	0·909	35	97·2	41	93·2	0·409
Fe	57	81·4	51	73·9	0·287	165	80·1	185	80·1	0·998	32	88·9	40	90·9	0·764
Vitamin A^†^	63	90·0	58	84·1	0·297	172	83·5	197	85·3	0·607	34	94·4	37	84·1	0·145
Vitamin B_1_	37	52·9	29	42·0	0·201	114	55·3	144	62·3	0·138	27	75·0	34	77·3	0·812
Vitamin B_2_	50	71·4	24	34·8	< 0·001^**^	159	77·2	188	81·4	0·278	34	94·4	41	93·2	0·816
Vitamin C	54	77·1	56	81·2	0·560	147	71·4	172	74·5	0·446	33	91·7	40	90·9	0·905
Niacin	33	47·1	16	23·2	0·003^*^	76	36·9	160	69·3	< 0·001^**^	24	66·7	35	79·5	0·193
Zn	64	91·4	64	92·8	0·772	168	81·6	221	95·7	< 0·001^**^	32	88·9	44	100	0·023^*^
Vitamin B_6_	60	85·7	62	89·9	0·456	159	77·2	218	94·4	< 0·001^**^	33	91·7	44	100	0·051
Vitamin B_12_	64	91·4	59	85·5	0·274	198	96·1	214	92·6	0·118	35	97·2	42	95·5	0·679
MAR	51	72·9	38	55·1	0·029^*^	151	73·3	191	82·7	0·018^*^	31	86·1	40	90·9	0·499

*P* values were calculated using *χ* ^2^ test.

* *P* < 0·05.

** *P <* 0·001.

† Retinol activity equivalent (RAE), 1 RAE = 1 µg retinol, 12 mg *β*-carotene, 24 mg α-carotene or 24 mg *β*-cryptoxanthin.

### Nutrient adequacy ratio and correlation with dietary diversity score

Correlations between the NAR and MAR of all selected nutrients and the DDS across age groups during the rainy and dry seasons are presented in Table 5. Most significant moderate to strong positive correlations between DDS and both individual NAR and MAR were observed exclusively in the dry season across all age groups, particularly for NAR of energy, vitamin B_2_, vitamin C and niacin (*r* = 0·418–0·691, *P* < 0·001). Across both seasons, older people exhibited a greater number of significant moderate to strong correlations (*r* = 0·446–0·734, *P* < 0·001) between DDS and nutrient adequacy compared with the other age groups, particularly for the NAR of energy, protein, Ca, Fe, vitamin B_2_ and the MAR. In contrast, only moderate correlations were observed between DDS and the NAR for energy (*r* = 0·418–0·469, *P* < 0·001) in school-age children and between DDS and the NAR for protein (*r* = 0·442–0·559, *P* < 0·001) in working-age people during both seasons.

**Table 5:** Nutrient adequacy ratios (NAR) and mean adequacy ratio (MAR) according to dietary diversity score (DDS)

Nutrient	DDS					
	School-age children		Working-age people		Older people	
	Rainy (*n* 70)	Dry (*n* 69)	Rainy (*n* 206)	Dry (*n* 231)	Rainy (*n* 36)	Dry (*n* 44)
Energy, kcal	0·469^**^	0·418^**^	0·241^**^	0·497^**^	0·452^**^	0·682^**^
Protein, g	0·505^**^	0·365^**^	0·442^**^	0·559^**^	0·515^**^	0·734^**^
Ca, mg	0·382^**^	0·387^*^	0·331^**^	0·464^**^	0·644^**^	0·529^**^
Fe, mg	0·290^*^	0·243^*^	0·249^**^	0·405^**^	0·585^**^	0·720^**^
Vitamin A^†^, µg	0·406^**^	0·217	0·248^**^	0·347^**^	0·454^**^	0·286
Vitamin B_1_, mg	0·217	0·279^*^	0·245^**^	0·355^**^	0·345^*^	0·580^**^
Vitamin B_2_, mg	0·386^*^	0·443^**^	0·363^**^	0·506^**^	0·565^**^	0·625^**^
Vitamin C, mg	0·196	0·510^**^	0·102	0·443^**^	0·174	0·610^**^
Niacin, mg	0·270	0·490^**^	0·146^*^	0·420^**^	0·146	0·484^**^
Zn, mg	0·152	0·393^**^	0·204^*^	0·382^**^	0·151	0·557^**^
Vitamin B_6_, mg	0·130	0·244^*^	0·117	0·220^**^	−0·005	0·299^*^
Vitamin B_12_, µg	0·309^*^	−0·023	0·514^**^	0·286^**^	0·340^*^	0·154
MAR	0·058^**^	0·466^**^	0·330^**^	0·521^**^	0·446^**^	0·691^**^

Spearman correlation coefficients were calculated to assess the association between DDS and each of the NAR and the MAR.

* *P* < 0·01.

** *P* < 0·001.

† Retinol activity equivalent (RAE), 1 RAE = 1 µg retinol, 12 mg *β*-carotene, 24 mg α-carotene or 24 mg *β*-cryptoxanthin.

## Discussion

Our study has revealed that the Karen people residing in rural western Thailand are facing a triple burden of malnutrition wherein overweight/obesity, undernutrition and micronutrient deficiencies coexist despite the year-round availability of diverse traditional food sources. To our knowledge, this study is also the first to examine the effects of seasonality on DDS and FVS across different age groups among Karen communities. School-age children had higher DDS compared with working-age and older people, largely due to the higher intake of dairy products and non-alcoholic beverages. However, significant seasonal variation was observed only in FVS among school-age children, while DDS remained consistent across seasons and age groups. Moderate to strongly positive correlations were observed exclusively in the dry season across all age groups between DDS and the NAR for energy, vitamin B_2_, vitamin C, niacin and MAR (*r* = 0·418–0·691, *P* < 0·001). Notably, across both seasons, older people demonstrated a higher number of significant moderate to strong correlations between DDS and NAR than the other age groups, particularly for the NAR of energy, protein, Ca, Fe, vitamin B_2_ and the MAR (*r* = 0·446–0·734, *P* < 0·001). This finding highlights a critical nutritional gap for these communities.

Most notably, the average energy intake among participants, particularly older people, was relatively low (< 1000 kcal/d) and at the lower end or below the national median reported among Thai older adults (male: 1004·5–1242·7 kcal/d; female: 928·5–1045·3 kcal/d)^(40)^. Additionally, over 35 % of older participants were underweight, compared with 10·8 % in the national survey of Thai adults aged over 60 years^(29)^. While energy intake is not entirely correlated with BMI, factors such as physical activity, nutrient density and BMR must also be considered^(41)^. Nonetheless, the high prevalence of underweight and low FVS among older adults in this study suggests limited dietary diversity, which may contribute to insufficient energy intake.

Previous research documented that the Karen living in Sanephong village and included in the present study relied on more than 387 traditional food species sourced from natural environments and cultivation, with many indigenous foods identified as rich sources of vitamins and minerals^(11)^. However, the global transition towards Western dietary patterns has been linked to rising malnutrition among indigenous populations^(42,43)^, as reflected in the nutritional challenges observed within the studied Karen communities. This trend is also consistent with the broader malnutrition situation in Thailand^(4,44)^.

To evaluate dietary patterns in the Karen community across seasons, this study adapted the DDS guidelines endorsed by the FAO^(16)^ and classifications from the Fifth Thai National Health Examination Survey^(29)^. The food groups were modified to reflect the Karen socioecological context. Within each season, significant differences in DDS and FVS were observed among the three age groups, with school-age children showing higher DDS, primarily due to greater consumption of dairy products and non-alcoholic beverages. However, no significant differences in DDS were observed between the two seasons in all three age groups. Only the FVS of school-age children significantly differed between rainy and dry seasons. This contrasts with prior studies showing clear seasonal effects on DDS^(45,46)^. Although seasonality has been widely explained as a factor influencing dietary diversity and nutrient adequacy, it may not be generalisable to all populations in different communities with distinct socioecological factors, such as socio-economic status, ecosystem, topography, climate, cultural background and traditional wisdom^(46)^.

Although age groups differed in DDS and FVS within seasons, overall FVS ranged only from 5·67 to 8·17, which is relatively low compared with previous reports of 14 to 20^(19)^. Traditional meal patterns remain prevalent, with most Karen families consuming two meals daily, typically consisting of chilli paste with vegetables, soups, fried foods and curries^(11)^. Furthermore, a previous study by Chotiboriboon *et al.* found Fe-rich food sources in a Karen community, including a type of snail called *Khlu-mi* and *Bai-ma-ngua* (*Citrus medica* L. var. medica) (dark green leaves)^(11)^. However, according to local people, *Khlu-mi* is now rare due to flooding in the villages in 2018. This exemplifies how natural disasters – linked to climate change and described by WHO as the ‘defining issue’ for 21st-century public health^(47)^ – threaten traditional food systems and warrant further study.

Interestingly, although vegetables were commonly consumed across all age groups, the intake of vitamins A and C remained insufficient, most likely attributable to both suboptimal portion sizes and high-heat cooking methods^(48)^. For instance, vegetables high in vitamins A and C, such as *Pak-man-mu* or *Le-khawng-du* (*Gnetum nemon* L. var. tenerum Markr.) (dark green leaves), *Yawd-fak-kao (Momordica chochinchinensis* Spreng) (young leaves) and *Bai-ma-ngua* (*Citrus medica* L. var. *medica*) (dark green leaves), were regularly included in meals but may not have been consumed in sufficient quantities to meet daily nutrient requirements. Similarly, *Bai-ma-ngua* (*Citrus medica* L. var. *medica*), a rich source of vitamins C and A, Fe and Ca, was available but did not appear to contribute adequately to overall nutrient intake. Moreover, the widespread practice of boiling vegetables – particularly among older adults who tend to retain traditional food preparation habits – likely further reduces the bioavailability of water-soluble and heat-labile nutrients, including vitamin C and some B vitamins.

The DDS in this study used a threshold of ≥ 15 g/d to count food items, reflecting both quantity and quality of intake. This approach underscores that seasonal variation in food availability may not be the primary driver of nutritional status in these Karen communities, as a variety of traditional foods – including rice, vegetables, fruits and fish – are available throughout the year. Instead, other factors such as socio-economic status, cultural norms, environmental changes and cooking practices appear more influential in shaping dietary quality and nutrient adequacy^(46)^.

These findings underscore the need to preserve traditional food systems and cultural dietary practices while addressing persistent nutrient gaps through culturally appropriate interventions. The vulnerability of indigenous food sources to climate change and environmental disruptions further threatens food security and health outcomes. Moreover, socio-economic transitions – such as increased wage labour, government employment and labour migration – have contributed to higher household incomes and food purchasing expenditures compared with earlier reports^(11)^, marking a shift from a traditional to a mixed food system^(49)^. This transition may reduce reliance on diverse indigenous foods and increase dependence on market-based products, with potential implications for diet quality and nutritional status. Future research should explore the detailed contribution of traditional *v*. market foods to dietary intake, as well as investigate strategies to promote nutrient retention during food preparation and to enhance access to diverse, nutrient-rich foods year-round.

### Strengths and limitations

A key strength of this study lies in its investigation of nutrient adequacy and dietary diversity across different age groups – school-age children, working-age people and older people – within Karen communities and across two distinct seasons. Notably, one of the two Karen villages included in this study was also examined in 2009^(11)^, allowing for comparative insights into the ongoing transition of traditional food systems. Furthermore, the use of the INMUCAL software, which incorporates a database of Thai foods along with selected Karen food items, provided a culturally relevant and context-specific approach to estimating dietary intake.

However, several limitations may have impacted this study and should be kept in mind for future research. First, dietary intake data were collected using a single-day 24-h dietary recall, which may not fully capture individuals’ habitual diets or the typical variety of foods consumed. Second, during the rainy season – coinciding with the beginning of the planting season – many working-age people and some school-age children accompanied their families to upland rice swidden areas located far from the village, often for several days or weeks. This seasonal migration resulted in a lower number of participants than originally calculated for the sample size. Third, most school-age children were interviewed on weekends due to difficulties in conducting interviews after school on weekdays, such as continuous rainfall during the rainy season and limited lighting, which relied on solar panels. In contrast, dietary interviews during the dry season were more evenly distributed between weekdays and weekends. This discrepancy may have influenced the reported frequency of certain food group consumption, especially milk, which is more likely to be consumed at school. Finally, limitations in the INMUCAL software database – particularly for Zn, vitamin B_6_ and vitamin B_12_ – may have led to underestimations of actual nutrient intakes^(31)^.

### Conclusion

This study highlights the coexistence of traditional and modern dietary patterns among the Karen people and the nutritional challenges that arise from this transition. Despite year-round access to diverse food sources, micronutrient inadequacies – particularly among older people – persist, while increasing market food consumption, especially among school-age children, reflects the broader nutrition transition. Age- and season-specific variations in dietary variety underscore the complex interplay of cultural practices, food access and environmental factors. These findings emphasise the need for culturally sensitive interventions that strengthen traditional food systems, improve food preparation practices to retain nutrient quality and support sustainable strategies to enhance dietary diversity and nutritional adequacy across all age groups.

## Supporting information

Joompa et al. supplementary material 1 Joompa et al. supplementary material

Joompa et al. supplementary material 2 Joompa et al. supplementary material

Joompa et al. supplementary material 3 Joompa et al. supplementary material

## Supplementary material

For supplementary material accompanying this paper, visit https://doi.org/10.1017/S1368980025101225.
