# Supplementary material for: Seasonal variation in dietary diversity and food variety scores among an indigenous Karen population in western Thailand: a cross-sectional study
Source: Public Health Nutr. 2025 Sep 25;28(1):e168. doi: 10.1017/S1368980025101225 (PMC12722075; doi:10.1017/S1368980025101225)
Supplement: Joompa et al. supplementary material 1 — Joompa et al. supplementary material [file S1368980025101225sup001.docx]

**Supplementary Table 1.** Frequency and percentage of each group of participants consuming each food group

| **Food groups** | **School-age children, n (%)** | | | **Working-age people, n (%)** | | | **Older people, n (%)** | | |
| --- | --- | --- | --- | --- | --- | --- | --- | --- | --- |
|  | **Rainy**  **(n = 70)** | **Dry**  **(n = 69)** | ***p*** | **Rainy**  **(n = 206)** | **Dry**  **(n= 231)** | ***p*** | **Rainy**  **(n= 36)** | **Dry**  **(n = 44)** | ***p*** |
| grain, white root, and tuber | 70 (100) | 69 (100) | - | 204 (99.0) | 230 (99.6) | 0.497 | 36 (100) | 44 (100) | - |
| pulse, bean, nut, and seed | 1 (1.4) | 1 (1.4) | 0.992 | 8 (3.9) | 7 (1.4) | 0.625 | 4 (11.1) | 3 (6.8) | 0.499 |
| dairy and dairy product | 14 (20.0) | 43 (62.3) | < 0.001** | 3 (1.5) | 11 (4.8) | 0.050 | 1 (2.8) | 1 (2.3) | 0.886 |
| meat, poultry, and egg | 47 (67.1) | 64 (92.8) | < 0.001** | 118 (57.3) | 163 (70.6) | 0.004* | 12 (33.3) | 22 (50.0) | 0.134 |
| fish and seafood | 25 (35.7) | 24 (34.8) | 0.908 | 76 (36.9) | 115 (49.8) | 0.007* | 16 (44.4) | 17 (38.6) | 0.600 |
| vegetable | 58 (82.9) | 42 (60.9) | 0.004* | 201 (97.6) | 203 (87.9) | < 0.001** | 33 (91.7) | 35 (79.5) | 0.131 |
| fruit | 15 (21.4) | 14 (20.3) | 0.869 | 50 (24.3) | 56 (24.2) | 0.994 | 9 (25.0) | 12 (27.3) | 0.818 |
| fat and oil | 31 (44.3) | 18 (26.1) | 0.025* | 161 (78.2) | 47 (20.3) | < 0.001** | 12 (33.3) | 4 (9.1) | 0.007* |
| Non-alcoholic beverage | 23 (32.9) | 32 (46.4) | 0.103 | 33 (16.0) | 77 (33.3) | < 0.001** | 3 (8.3) | 12 (27.3) | 0.031* |

*p* values were calculated using Chi-Square test.

* *p* < 0.05, ** *p <* 0.001
