# Supplementary material for: Seasonal variation in dietary diversity and food variety scores among an indigenous Karen population in western Thailand: a cross-sectional study
Source: Public Health Nutr. 2025 Sep 25;28(1):e168. doi: 10.1017/S1368980025101225 (PMC12722075; doi:10.1017/S1368980025101225)
Supplement: Joompa et al. supplementary material 2 — Joompa et al. supplementary material [file S1368980025101225sup002.docx]

**Supplementary Table 2.** Median (25th and 75th percentiles) intake of energy and selected nutrients among participants by age group during the rainy season

| **Nutrient** | **School-age child** **(n = 70)** | | **Working-age people** **(n = 206)** | | **Older people** **(n= 36)** | |
| --- | --- | --- | --- | --- | --- | --- |
|  | **Nutrient content** | **%DRI*** | **Nutrient content** | **%DRI*** | **Nutrient content** | **%DRI*** |
| Carbohydrate:protein:fat, % | 63.1:10.6:24.9 |  | 72.7:10.7:15.6 |  | 76.1:9.9:9.3 |  |
| Energy, kcal | 1194.2 (885.0, 1551.0) | 73.4 (55.9, 94.4) | 1415.4 (1007.4, 1899.2) | 71.3 (52.3, 97.5) | 841.6 (627.5, 1225.2) | 50.1 (36.9, 71.1) |
| Carbohydrate, g | 180.3 (135.9, 219.5) |  | 245.1 (170.0, 336.9) |  | 163.3 (118.6, 195.9) |  |
| Fat, g | 30.5 (16.8, 47.1) |  | 24.1 (11.5, 40.7) |  | 9.6 (4.1, 24.6) |  |
| Protein, g | 30.2 (20.6, 47.8) | 93.3 (59.1, 132.8) | 39.2 (25.3, 55.0) | 70.6 (46.9, 99.5) | 23.2 (15.1, 38.6) | 45.5 (26.2, 77.2) |
| Calcium, mg | 156.2 (86.6, 244.6) | 16.5 (9.8, 28.6) | 184.7 (109.9, 341.9) | 26.4 (15.7, 48.9) | 90.8 (59.4, 207.9) | 13.0 (8.5, 29.7) |
| Iron, mg | 4.6 (3.1, 7.3) | 54.4 (31.8, 68.6) | 6.4 (4.1, 9.6) | 43.2 (25.2, 66.8) | 4.3 (2.2, 6.0) | 39.5 (21.2, 55.6) |
| Vitamin A^†^, µg | 99.5 (29.9, 218.9) | 21.3 (6.6, 44.3) | 161.5 (61.7, 327.2) | 23.4 (9.6, 54.2) | 60.7 (15.3, 213.3) | 9.5 (2.5, 31.6) |
| Vitamin B1, mg | 0.5 (0.3, 0.9) | 68.2 (41.2, 110.5) | 0.8 (0.5, 1.3) | 67.6 (40.6, 107.5) | 0.5 (0.3, 0.9) | 41.9 (27.3, 76.9) |
| Vitamin B2, mg | 0.4 (0.3, 0.7) | 58.6 (36.2, 87.5) | 0.6 (0.4, 0.9) | 47.3 (31.8, 72.2) | 0.4 (0.2, 0.5) | 28.1 (19.0, 41.8) |
| Vitamin C, mg | 17.2 (6.0, 36.3) | 32.3 (11.8, 65.2) | 31.4 (13.7, 77.1) | 33.5 (15.7, 82.2) | 25.3 (11.2, 47.3) | 26.4 (11.3, 52.9) |
| Niacin, mg | 8.4 (4.6, 11.7) | 80.6 (47.8, 107.7) | 12.7 (8.3, 19.1) | 86.7 (55.5, 126.9) | 8.7 (6.0, 11.9) | 57.8 (39.9, 78.1) |
| Zinc, mg | 2.9 (2.0, 4.2) | 36.6 (25.5, 54.1) | 4.7 (3.1, 6.8) | 46.1 (27.8, 72.2) | 2.7 (2.2, 4.4) | 28.0 (22.8, 44.5) |
| Vitamin B6, mg | 0.4 (0.2, 0.5) | 48.1 (29.5, 61.5) | 0.6 (0.4, 0.9) | 43.3 (27.8, 72.2) | 0.4 (0.3, 0.5) | 26.1 (18.5, 32.2) |
| Vitamin B12, µg | 0.3 (0, 0.9) | 18.8 (0, 51.7) | 0 (0, 0.5) | 0.2 (0, 21.3) | 0 (0, 0.3) | 0 (0, 12.5) |
| MAR | 0.6 (0.4, 0.8) |  | 0.5 (0.4, 0.8) |  | 0.4 (0.3, 0.5) |  |

*DRI, Dietary Reference Intake for Thais, 2020

†Retinol activity equivalent (RAE), 1 RAE = 1 µg retinol, 12 mg β-carotene, 24 mg, α-carotene, or 24 mg β-cryptoxanthin
