# Supplementary material for: Seasonal variation in dietary diversity and food variety scores among an indigenous Karen population in western Thailand: a cross-sectional study
Source: Public Health Nutr. 2025 Sep 25;28(1):e168. doi: 10.1017/S1368980025101225 (PMC12722075; doi:10.1017/S1368980025101225)
Supplement: Joompa et al. supplementary material 3 — Joompa et al. supplementary material [file S1368980025101225sup003.docx]

**Supplementary Table 3.** Median (25th and 75th percentiles) intake of energy and selected nutrients among participants by age group during the dry season

| **Nutrient** | **School-age child** **(n = 69)** | | **Working-age people** **(n = 231)** | | | **Older people** **(n= 44)** | |
| --- | --- | --- | --- | --- | --- | --- | --- |
|  | **Nutrient content** | **%DRI*** | | **Nutrient content** | **%DRI*** | **Nutrient content** | **%DRI*** |
| Carbohydrate:protein:fat, % | 55.3:14.2:30.9 |  | | 63.5:12.7:23.0 |  | 68.5:10.5:17.2 |  |
| Energy, kcal | 1240.5 (859.0, 1621.6) | 73.9 (60.1, 95.8) | | 1094.3 (812.1, 1650.8) | 56.5 (43.2, 83.3) | 781.8 (539.6, 1224.3) | 46.7 (31.2, 63.2) |
| Carbohydrate, g | 161.6 (117.1, 216.8) |  | | 177.3 (125.2, 242.4) |  | 140.5 (100.0, 204.3) |  |
| Fat, g | 45.6 (30.5, 55.8) |  | | 26.4 (13.7, 51.4) |  | 13.1 (7.0, 25.6) |  |
| Protein, g | 44.1 (31.4, 57.2) | 123.9 (96.1, 163.1) | | 34.3 (22.6, 54.3) | 61.2 (42.1, 95.9) | 21.4 (13.1, 33.6) | 39.7 (24.7, 61.7) |
| Calcium, mg | 349.5 (171.6, 535.9) | 38.3 (17.2, 57.4) | | 191.4 (118.7, 379.3) | 27.3 (17.0, 51.8) | 125.6 (76.1, 220.8) | 18.0 (10.9, 31.5) |
| Iron, mg | 5.4 (3.9, 8.2) | 47.1 (36.5, 77.4) | | 5.3 (3.2, 8.6) | 34.7 (18.9, 64.1) | 2.8 (1.7, 5.8) | 27.0 (15.5, 52.3) |
| Vitamin A^†^, µg | 171.8 (97.1, 282.6) | 38.7 (19.5, 52.6) | | 129.4 (40.3, 337.2) | 20.4 (6.6, 51.6) | 63.4 (24.7, 208.7) | 9.4 (3.6, 31.5) |
| Vitamin B1, mg | 0.8 (0.4, 1.7) | 108.2 (58.6, 205.7) | | 0.7 (0.4, 1.1) | 62.0 (36.2, 95.6) | 0.5 (0.2, 0.8) | 43.1 (20.4, 71.0) |
| Vitamin B2, mg | 0.8 (0.5, 1.2) | 98.8 (57.7, 138.8) | | 0.4 (0.2, 0.7) | 34.7 (19.0, 59.7) | 0.3 (0.2, 0.5) | 21.5 (11.9, 46.0) |
| Vitamin C, mg | 16.0 (8.1, 37.1) | 34.0 (15.4, 68.1) | | 31.5 (13.1, 66.1) | 34.9 (15.0, 76.0) | 25.0 (7.8, 53.7) | 25.7 (8.1, 58.3) |
| Niacin, mg | 16.6 (7.8, 23.6) | 143.7 (84.8, 221.2) | | 8.3 (5.7, 13.2) | 57.3 (40.2, 88.6) | 7.1 (4.1, 10.8) | 46.3 (28.1, 72.1) |
| Zinc, mg | 3.2 (2.1, 4.3) | 38.7 (25.0, 50.2) | | 2.8 (1.8, 4.3) | 28.7 (18.6, 43.6) | 2.4 (1.3, 3.1) | 24.8 (13.6, 32.3) |
| Vitamin B6, mg | 0.3 (0.2, 0.5) | 35.5 (20.2, 56.9) | | 0.4 (0.2, 0.6) | 27.1 (14.3, 42.3) | 0.3 (0.2, 0.5) | 20.2 (10.6, 28.2) |
| Vitamin B12, µg | 0.4 (0, 0.8) | 23.9 (0, 50.4) | | 0 (0, 0.5) | 0 (0, 21.7) | 0 (0. 0.3) | 0 (0, 11.6) |
| MAR | 0.7 (0.6, 1.0) |  | | 0.4 (0.3, 0.7) |  | 0.3 (0.2, 0.5) |  |

*DRI, Dietary Reference Intake for Thais, 2020

†Retinol activity equivalent (RAE), 1 RAE = 1 µg retinol, 12 mg β-carotene, 24 mg, α-carotene, or 24 mg β-cryptoxanthin
